# Dual-Attitude Decision-Making Processes of Construction Worker Safety Behaviors: A Simulation-Based Approach

**DOI:** 10.3390/ijerph192114413

**Published:** 2022-11-03

**Authors:** Man Zhou, Xiancong Chen, Lei He, Franck Aristide Kiswendsida Ouedraogo

**Affiliations:** 1 School of Civil Engineering, Central South University, Changsha 410075, China; 2 School of Civil Engineering, Wuhan University, Wuhan 430072, China; 3 School of Public Administration, Central South University, Changsha 410075, China; 4 Center for Social Stability Risk Assessment, Central South University, Changsha 410017, China

**Keywords:** safety behavior, dual-attitude theory, system dynamics modeling, meta-analysis

## Abstract

Workplace accidents are of great concern in the construction industry. Most of those accidents are caused by unsafe behavior in the workplace. Many previous studies have analyzed the causes of workers’ unsafe behaviors, but few have investigated workers’ feelings of insecurity from the perspective of systematic psychological theory. This study developed an attitude–behavior–intervention feedback loop mechanism of construction workers and used the dual-attitude theory to explain the occurrence mechanisms of unsafe behavior. Using this mechanism, an active-intervention system-dynamics model and a passive-intervention system-dynamics model were designed and simulated. The coefficient of the system dynamics equation in the simulation model involved meta-analysis to combine the correlation coefficients of existing studies, which increased the sample size and improved the statistical test efficiency. The results show that an implicit safety attitude has a more significant impact on safety behavior, and the effect of an active intervention is stronger than that of a passive intervention. Based on these results, this paper presents some feasible suggestions to reduce the probability of unsafe worker behaviors occurring.

## 1. Introduction

The number of accidents in the construction industry has increased considerably in recent years, and the casualty rate in the construction industry is the highest of almost all industries in the world [1,2]. A report by the International Labor Organization stated that the fatalities and disabling injuries in the construction industry were three times higher than in other sectors in 2016. Construction safety has also become a focus of social attention. According to Heinrich’s classic domino theory [3], the leading causes of accidents are unsafe conditions and actions in the workplace; he pointed out that more than 88% of accidents are caused by unsafe behavior. Analyzing the reasons behind the unsafe behavior of workers on construction sites enables the regulation of their behavior and the adoption of effective management measures to reduce accidents. On this basis, construction safety management has been committed to two aspects: behavior-based safety (BBS) and the theory of planned behavior (TPB).

The existing BBS approaches adopt a top-down process in which managers are responsible for observing worker behavior, making positive or negative interventions, and obtaining feedback [4]. For example, Zaira and Hadikusumo [5] performed exploratory factor analysis and structural equation modeling to identify the most vital intervention-related safety practices; the results suggested that technical interventions positively influenced management and human interventions. Mohammadfam et al. [6] presented a model for managing and improving the safety behavior of employees using a Bayesian network approach. The results indicated that instantaneous improvement of a supportive environment and employee participation are the best strategies to reach a high proportion of safety behavior in the workplace. This approach was based on the theory of behaviorism, which concentrates on observable behavior rather than an unobservable attitude toward safety [7]. The existing BBS methods violate the original intention of BBS because they do not consider the influence of internal factors such as safety awareness and safety knowledge, causing confrontations between workers and managers [8,9]. As a result, it has been observed that, in most cases, the effect of current BBS approaches is minimal and worker behavioral safety performance decreases once external intervention programs are cancelled [10]. This phenomenon has become a significant obstacle to the further development and promotion of BBS practice [11,12].

Therefore, some scholars have proposed the theory of planned behavior (TPB) [13], which is more appropriate for understanding worker safety behavior. The TPB focuses on the psychological dimension, emphasizing the effect of behavioral intentions on behavior occurrence [14], such that those workers who develop positive attitudes toward safe behavior are more willing to work safely [15]. For example, Peng and Chan employed the TPB to predict the safety behavior of older construction workers and found that subjective norms were the strongest predictors of safety behavior [16]. More recently, Mohajeri et al. analyzed the moderating effects of the TPB on safety habit variables and showed that when the level of safety habits was high, the correlation between safe and unsafe behavior was strongly negative [17]. In addition, many researchers have extended the TPB by including habits that directly impact safety behavior [18,19].

Some attempts to explain the mechanisms behind unsafe behaviors based on mental factors are presented in the literature. Most studies have focused on the static correlation between discrete factors, and few scholars have used systematic psychological theories to explain the mechanisms behind unsafe behavior. It is challenging to determine the psychosocial decision-making process that influences an individual to choose to perform safe or unsafe behavior; the decision-making process influencing safe behavior needs more attention from both systematic and dynamic perspectives.

This study aimed to gain a more comprehensive understanding of the relationship between attitude and behavior to better explain worker safety behavior. This paper primarily discusses the ontology of safety behavior and proposes an attitude–behavior–intervention feedback loop mechanism, the content of which is enriched via the psychological dual-attitude theory.

Two system dynamics simulation models were conducted. The results guide the safety management suggestions that are provided and are based on theoretical analysis and system dynamics modeling analysis. These suggestions may prevent and control the unsafe behavior of construction workers. The coefficient of the system dynamics equation is the correlation coefficients of each path of the theoretical mechanism. In order to improve the generalizability of research conclusions, this study employed a meta-analysis to combine the correlation coefficients of existing studies, rather than questionnaire surveys [20] and expert ratings [21] which are commonly used. This analysis method increases the sample size and improves the statistical test efficiency [22,23].

## 2. Dual-Attitude Theoretical Model

### 2.1. Attitude–Behavior–Intervention Feedback Loop Mechanism

**Figure 1:**
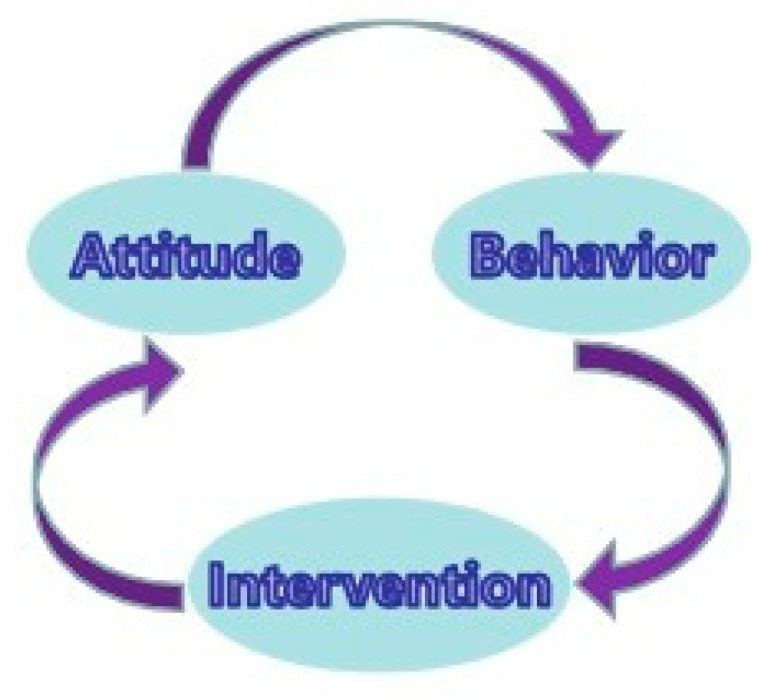
Attitude–behavior–intervention feedback loop.

The TPB model is an open theoretical framework that can incorporate new factors to improve the interpretation of human behavior. Previous studies have focused on explaining the factors influencing unsafe behavior by establishing the relationship between attitude, intention, and behavior [24]. In contrast, this study treated attitude, management, and behavior as a cyclic feedback process, as shown in Figure 1. If worker attitude directly affects safety behavior, managers will take intervention measures by collecting unsafe worker behaviors. These interventions will change workers’ attitudes, which, in turn, will influence subsequent safety behavior. The collected safety behaviors can be returned to the manager for subsequent interventions. The relationship between them will continue to improve via the cycle.

Next, we expand the feedback loop structure and introduce the theory of dual attitude to analyze the mechanisms behind the unsafe behavior of construction workers.

### 2.2. A Dual-Attitude Theoretical Model Based on Construction Workers’ Unsafe Behavior

According to psychological research, attitude refers to an individual evaluation and behavioral tendency towards a particular object. It is often assumed that attitude informs deliberate and goal-oriented reasoning regarding action, which translates into intended behavior [25]. In the construction field, safety attitude refers to workers’ cognition, emotional tendency, and behavioral response to various factors in workplace safety.

Attitude is not merely a singular psychological tendency towards an object; this is especially true in a complex social environment. In addition to those attitudes that can be realized or have been shown, people may have some attitudes of which they are unaware. According to the dual-attitude model theory, people have two different attitudes towards the same object simultaneously: one is the explicit attitude that people can realize, and the other is the unconscious and habitual implicit attitude. 

To clarify, people actively remember the accidents and risk factors involved in incidents that threaten their safety and undertake corresponding safety attitude, which can be retained in their memory for a long time. When they see the same harm in their workplace accidents or similar risk factors, this safety attitude is automatically activated and is rarely controlled by consciousness. The stored safety attitude gradually forms an implicit safety attitude which people themselves do not perceive. Moreover, people can construct new attitudes according to their current social situations, behaviors, thoughts, and emotions. Such new attitudes, which can be extracted and easily expressed in words, are formed based on some internal rationale, such as adapting to a production organization or striving to avoid accidents. According to the dual-attitude model theory, this sort of attitude that can be formed or changed immediately according to people’s thoughts, behavior, and environment is called explicit attitude.

**Figure 2:**
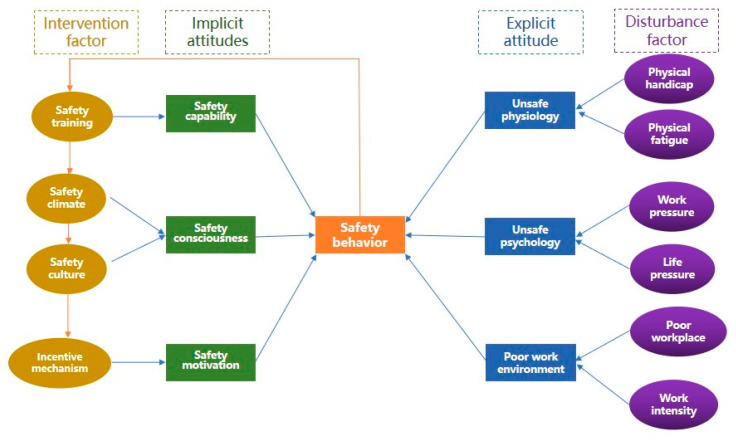
A dual-attitude theoretical model based on construction workers’ unsafe behavior.

Workers in construction have an automatically activated implicit safety attitude and an explicit safety attitude sensitive to environmental information. Combined with the cyclic feedback mechanism between attitude, behavior, and intervention mentioned above, this study establishes a theoretical model of dual safety attitude based on construction workers’ unsafe behavior to explore the relationship between safety attitude, behavior, and management, as shown in Figure 2.

On the one hand, behavior may be triggered by explicit processes. Along this path, explicit factors promote the emergence of explicit safety attitudes, which facilitates the development of behavioral intentions, which drive behavior. In the above theoretical model, explicit factors affecting safety attitudes include unsafe physiology, unsafe psychology, and a poor working environment. Physical handicap and fatigue, work and life pressure, poor workplace, and work intensity are the disturbance factors of unsafe physiology, unsafe psychology, and a poor working environment, respectively. First, working while physically ill itself is an unsafe behavior. Physical handicaps such as cold, fever, and fatigue could make people suffer from dizziness, backache, and other physiological symptoms of decline. Workers may feel tired and powerless, resulting in unsafe behavior. Second, intense work and life pressure can make people anxious, depressed, and irritable; thus, workers may exhibit unsafe behavior, causing workplace accidents. Third, a poor workplace such as working high above the ground and a high work intensity make people fearful, which leads them to undertake unsafe behavior. Under the influence of external factors in the production process, workers will establish a safe-aware and self-controlled safety attitude based on certain reasons or needs to adapt to the production organization, avoid accidents, and avoid violating safety management regulations. Although explicit safety attitudes can be stored in the memory system, they do not have characteristics of automatic activation and rapid response, as opposed to the implicit safety attitude. Explicit attitudes require behavioral motivation or mental energy drawn from the memory system, or which are formed immediately.

On the other hand, behavior can also be driven by implicit attitudes. Implicit attitudes, which are automatically activated when exposed to cues related to the object of the attitude (for example, the discovery of a broken leaky wire), can directly drive one’s behavior without processing through one’s conscious thought paths and developmental intentions. In the above theoretical model, the implicit factors affecting implicit safety attitude include safety capability, safety consciousness, and safety motivation. First, safety capability refers to workers’ knowledge, skills, and experience in production, safety and accident prevention, which is the basis for them to implement production plans safely and effectively. When the workers’ safety capability is stronger, they can be more confident and calmly engage in safety production. Second, safety consciousness refers to the “human body’s own understanding of safety issues” [26]. Concerning construction workers, focus on psychological protection and vigilance against adverse working conditions may affect health and safety. Thirdly, safety motivation includes conformity, speculative motivations, habit, and lucky motivations. Conformity means behaving like others or doing what others do; speculative motivation refers to blindly saving time and effort in order to speed up progress or reduce physical loss; habit refers to the habitual behavior of individuals; and lucky motivation refers to a worker’s belief that an accident could not happen to them. In the process of construction, unsafe motivations of construction workers are common, and some seemingly efficient decisions may become a substantial hidden danger to safety. People’s demand for safety comes from their physiological desire, so they have innate demand for safety, which leads them to attach great importance to self-protection and maintenance of their safety state. At the same time, with the growth of the population and the change in the surrounding environment, although the relevant memories may have partially disappeared, the formed implicit safety attitude will affect people’s behavior in the long term. Due to their long-term existence in the human memory and cognitive system, implicit safety attitudes are highly stable and difficult to change. Managers can improve workers’ implicit safety attitudes through some intervention measures such as safety training, safety clime communication, safety culture publicity, and improving incentive mechanisms.

The ultimate attitude of the individual depends on the strength of the two attitudes. In general, implicit safety attitudes are more easily activated. Implicit attitudes are automatically triggered; therefore, when facing the same object, even if the worker activates and displays the corresponding explicit safety attitude based on some factors, the implicit safety attitude they have already formed could potentially affect their safety behavior and thought. However, the situation on safety production sites is complicated, and workers often face conflicting subjective situations in their work. In order to deeply understand the activation of safety attitudes in workers with dual safety attitude in real life, we designed a simulation experiment.

## 3. Methodology

### 3.1. Framework

In this study, a method based on system dynamics and discrete event simulation is proposed to simulate the occurrence mechanism of the unsafe behavior of construction workers. There have been some studies on construction workers’ safety behaviors using system dynamics, but one-way paths between influencing factors and safety behaviors were exclusively assessed, rather than the attitude–behavior–intervention feedback loop proposed in this paper. Moreover, discrete event simulation is first introduced to study it, which allows for more flexible controls of the initiation and termination of some conditions such as interventions and forgetting mechanisms.

**Figure 3:**
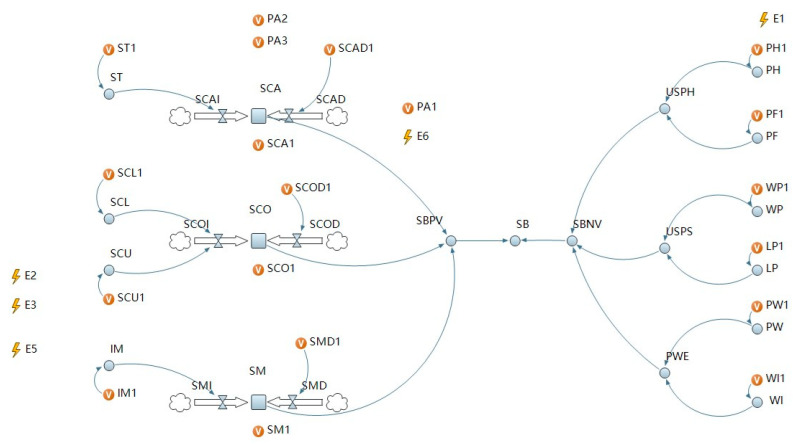
The active intervention simulation model of unsafe behavior of construction workers. ST = Safety training, SCL = Safety climate, SCU = Safety culture, IM = Incentive mechanism, SCA = Safety capability, SCO = Safety consciousness, SM = Safety motivation, SB = Safety behavior, USPH = Unsafe physiology, USPS = Unsafe psychology, PWE = Poor work environment, PH = Physical handicap, PF = Physical fatigue, WP = Work pressure, LP = Life pressure, PW = Poor workplace, WI = Work intensity, PA *i* = Parameter *i* (*i* = 1,2,3), E *i* = Event *i* (*i* = 1,2,3,4,5,6,7), SBPV = the positive values of SB, SBNV = the negative values of SB, SCAI (SCOI, SMI) = the increment of SCA (SCO, SM, SB), SCAD (SCOD, SMD) = the decrement of SCA (SCO, SM, SB)).

**Figure 4:**
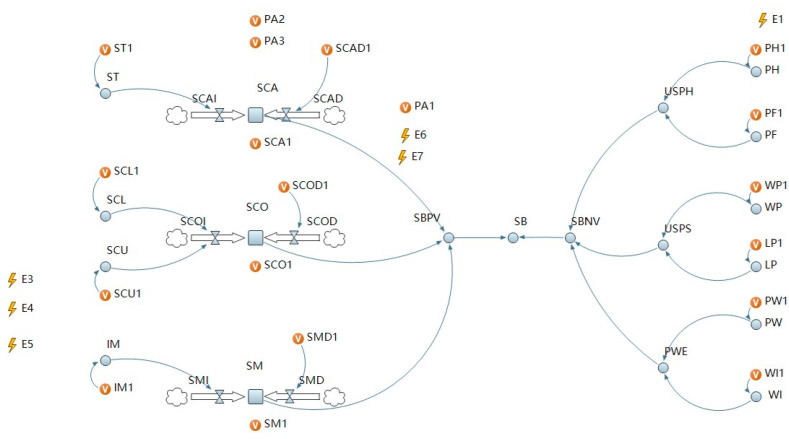
The passive intervention simulation model of unsafe behavior of construction workers.

Firstly, we established the causal feedback structure of system dynamics. According to the path relationships between the influencing factors in the dual-attitude theoretical model based on construction workers’ unsafe behavior and the characteristics of the stock, flow, and dynamic variables in the system dynamics model, the stock, flow, and dynamic variables of the simulation model and the system dynamics equation of the three were defined in turn. The system dynamics equation parameters were the theoretical model’s correlation coefficients. Secondly, in consideration of the intervention conditions and the attenuation of implicit attitude over time, discrete events were used to control them, and the active intervention simulation model and the passive intervention simulation model were designed, as shown in Figure 3 and Figure 4. Then, in order to obtain the coefficients of the system dynamics equation, we introduce how to combine the correlation coefficients of previous studies with meta-analysis.

### 3.2. Simulation Model Construction

The variables in the model were qualitative; therefore, they were measured in dimensionless units and represented by the unit “1”. The safe behavior value was recorded daily as a real-time dynamic variable. Its value is affected by the positive effect value and negative effect value of the day, and its system dynamics equation can be expressed as:

SB = SBPV − SPNV(1)

The value of SBPV is affected by implicit factors, and its system dynamics equation can be expressed as:

SBPV = X_1_ ×SCA + X_2_ × SCO + X_3_ × SM + σ_1_ (2)

The value of SPNV is affected by explicit factors, and its system dynamics equation can be expressed as:

SBNV = X_4_ × SCA + X_5_ × SCO + X_6_ × SM + σ_2_ (3)

where X*_i_* (*i* = 1, 2, … *n*) is the parameter of the system dynamics equation and σ*_i_* (*i* = 1, 2 … *n*) is various error terms, the same as below. 

System dynamics is based on feedback loops; therefore, it is insensitive to most non-critical parameters in the model. As long as the parameter estimation is within a reasonable range, there will be little or no deviation in the model results [27]. Therefore, the influence of the error term σ*_i_*(*i* = 1, 2, … *n*) was not considered in this study, and its value was set to 0, the same as below.

Next, we analyzed the disturbance factors. We defined physical handicap as a dynamic variable because a worker’s health status varies daily. It is real-time rather than cumulative. The same applies for other disturbances such as changing work sites, less work completed that day, etc. Here, we assigned a value to it every day by cyclic Event 1 (see Appendix A for the properties of Event *i* (*i* = 1, 2, 3, 4, 5, 6, 7)). In addition, considering that variables of the system dynamics module and variables in discrete events cannot be used together, another variable was introduced under the agent module and made equal. It can be expressed as:

PH = PH1(4)

ST1, SCL1, CSU1, IM1, SCA1, SCAD1, SCO1, SCOD1, SM1, SMD1, PF1, WP1, LP1, PW1, and WI1 are the same.

Explicit factors directly affected by disturbance factors are also real-time dynamic variables, and the system dynamics equations between them can be expressed as:

USPH = X_7_ × PH + X_8_ × PF + σ_3_ (5)

USPS = X_9_ × WP + X_10_ × LP + σ_4_ (6)

PWE = X_11_ × PW + X_12_ × WI + σ_5_ (7)

Considering the impacts of cost, schedule, and other factors, safety training and other intervention factors cannot be performed frequently. Input values are only available when the interventions occur; thus, we defined them as real-time dynamic variables. Two interventions were established in this study, and the duration of the intervention was one day. One was active intervention, a periodic intervention, i.e., where the managers intervened in the system once a month on the first day. The second kind was passive intervention, which was an irregular intervention. When the safety behavior value of the preceding month was less than 0.2, this meant that the safety attitude of workers was poor and the frequency of unsafe behavior was high, and the managers intervened in the system once on the first day of the following month.

In the active intervention model, we assigned values to the intervention factors by cyclic Event 2 and controlled the duration of the intervention by cyclic Event 3.

In the passive intervention model, we assigned values to the intervention factors by cyclic Event 4 and controlled the duration of the intervention by cyclic Event 3.

Each of the implicit factors increased with an input value when the interventions were triggered. We considered that their values during the two interventions were not invariable and would decay with time until the next intervention was triggered at a new rate. Therefore, they are cumulative stocks. Each implicit factor had corresponding increases and decreases, and their system dynamics equations are:

SCA = SCAI − SCAD(8)

SCO = SCOI − SCOD(9)

SM = SMI − SMD(10)

The values here may be greater than 1; therefore, we set cyclic Event 5 to control it.

The system dynamics equations between the increase in each implicit factor and its corresponding intervention factors can be expressed as:

SCAI = X_13_ × ST + σ_6_ (11)

SCOI = X_14_ × SCL + X_15_ × SCU + σ_7_ (12)

SM = X_16_ × IM + σ_8_ (13)

In the initial stage of the system, considering that each unit had intervention measures such as pre-entry training, we took the value of the first intervention transformed by the above equation as the initial value of each implicit factor. The attenuation effect of each implicit factor had not yet come into play; therefore, the reduction in each implicit factor was 0. Therefore, the initial value of each implicit factor was equal to the increase in each implicit factor at this time.

For the decay of implicit factors, we introduced the formula of the memory forgetting curve [28] as the effect of their decay over time. It can be expressed as:

(14) $$R=e^{-\frac{t}{S}}$$

where *R* is the retrievability (a measure of how easy it is to retrieve a piece of information from memory), *S* is the stability of memory (which determines how fast *R* falls over time in the absence of training, testing, or other recall), and *t* is time.

Memory strength will increase after each intervention; therefore, a new round of slower decay is caused. It was supposed that the decay rate decreased by Z to the power of H (0 < Z < 1, H = 1, 2, … *n*), where H was the number of interventions. The reduction in each implicit factor was controlled by cyclic Event 6.

In the passive intervention model, the values of safety behavior could remain above 0.2 for a long time after several interventions (where the level of values is slightly greater than 0.2), during which there are no intervention measures, making workers relaxed and idle. In this regard, we introduced the complete forgetting mechanism. When there was no intervention for more than 60 consecutive days, its attenuation rate became the initial state, controlled by cyclic Event 7.

### 3.3. Meta-Analysis

As mentioned above, we used meta-analysis to merge the correlation coefficients of previous studies as parameters of the system dynamics equation in the simulation model.

In this study, meta-analysis recommended by Hunter and Schmidt [22] was used to merge effect values from the related literature. There are four steps in meta-analysis: (a) literature search; (b) literature selection; (c) literature coding; and (d) data analysis. To provide information on the estimate of population correlation, we report the study number (K); total sample size (N); uncorrected correlation value (r), which is sample size-weighted; the corrected correlation value (r_c_), which is sample size-weighted and reliability-corrected; and the 95% confidence interval for r_c_ (95%CI).

#### 3.3.1. Literature Search

Several retrieval techniques were adopted to ensure that the literature and samples included in the study were maximized, complete, and representative. First, considering timeliness, a span of 11.5 years was chosen for the literature search: from 1 January 2010 to 30 June 2021. Second, we searched the China National Knowledge Infrastructure (CNKI), the largest Chinese document database, and Web of Science, the largest international database. In addition, we used the keywords “construction” and “unsafe/safety behavior”. Finally, we have checked the literature we obtained manually to avoid missing some related studies.

#### 3.3.2. Literature Selection

After completing the literature search, we obtained 691 research articles and applied some inclusion and exclusion criteria to filter the existing literature further. First, the study had to be based on empirical research and needed to report the coding details, such as the sample size (n), correlation value (r), or other effect sizes (e.g., standardized multiple regression coefficients (β), *t* for *t*-test, F for F-test), which can be easily converted to r. Then, we excluded articles that: (a) presented qualitative or non-empirical research; (b) ambiguously defined their factors and presented relationships between factors; or (c) were less reliable or were of poor quality. Finally, 17 articles were retained for coding.

#### 3.3.3. Literature Coding

For each article, the coded information is listed as the article ID (number), author, publication date, title, article source, sample size (N), independent and dependent variables, and the correlation value r or other effect size between variables.

#### 3.3.4. Data Analysis

It was supposed that (X_1_, Y_1_), (X_2_, Y_2_), …, (X*_n_*, Y*_n_*) were randomly sampled from a population with a mean of (μ_1_, μ_2_), variance of ($\sigma_{1}^{2},\sigma_{2}^{2}$), and correlation value of ρ. For large samples, the following distribution was found [29]:

(15) $$r\sim N(\rho ,\frac{{\left(1-\rho^{2}\right)}^{2}}{n-1})$$

where *r* is the correlation value of the sample of two variables and ρ is the correlation value of the population of two variables. The actual correlation value of the population of two variables, ρ, was unknown; thus, it was generally estimated by the correlation value of sample *r*. The variance of the two values ν can be approximately estimated as [30]:

(16) $$\nu \approx \frac{{\left(1-r^{2}\right)}^{2}}{n-1}$$

The combination of correlation value *r* follows the general combination process of effect size [29,31].

It is assumed that all the correlation values are from the same population:

(17) $$\rho_{1}=\rho_{2}=\cdots \cdots =\rho_{k}=\rho$$

where ρ is unknown and can be estimated by the estimator $\hat{\rho }$:

(18) $$\hat{\rho }=\frac{\sum_{i=1}^{k}W_{i}r_{i}}{\sum_{i=1}^{k}W_{i}}$$

where *W_i_* is the weight of the Study *i*, and *r_i_* is the effect size of the Study *i*.

Variance reflects whether the measurement index is accurate or not, so the mutual variance of each study $\sigma_{\left(\rho_{i}\right)}^{2}$ can be used to represent the weight *W_i_* of each study:

(19) $$W_{i}=\frac{1}{\sigma_{\left(\rho_{i}\right)}^{2}}$$

$\sigma_{\left(\rho_{i}\right)}^{2}$ is unknown and can be estimated by the variance of each sample $\nu_{i}$; then, the estimator $\hat{\rho }$ can be expressed as:

(20) $$\hat{\rho }=\frac{\sum_{i=1}^{k}r_{i}/\nu_{i}}{\sum_{i=1}^{k}1/\nu_{i}}$$

where *r_i_* is the correlation value of Study *i*.

Its variance, $\mathrm{var}(\hat{\rho })$, can be expressed as:

(21) $$\mathrm{var}(\hat{\rho })\approx \frac{1}{\sum 1/\nu_{i}}$$

The significance, *Z*, of the combined estimator, $\hat{\rho }$, can be tested as:

(22) $$Z=\frac{\left|\hat{\rho }\right|}{\sqrt{\mathrm{var}(\hat{\rho })}}=\left|\hat{\rho }\right|\sqrt{\sum_{i=1}^{k}\frac{1}{\nu_{i}}}$$

Then, the confidence interval of the actual value of the combined estimator, $\hat{\rho }$, is:

(23) $$\hat{\rho }-Z_{\alpha /2}\sqrt{\mathrm{var}(\hat{\rho })}\leq \rho \leq \hat{\rho }+Z_{\alpha /2}\sqrt{\mathrm{var}(\hat{\rho })}$$

## 4. Simulating and Result Analysis

### 4.1. Initializing Parameters

Although we described the construction idea of the simulation model above, we also needed to initialize the relevant parameters before simulating. INITIAL TIME = 0, FINAL TIME = 720, TIME STEP = 1 were set, and the UNIT for TIME was day. Thus, we simulated 2 years (system TIME 1 month = 30 days) and recorded the safety behavior value of workers once per day.

In cyclic Event 1, considering that workers with excessive and severe physical handicaps cannot engage in safe production, the value of other disturbance factors was randomly taken between 0 and 1, except for physical handicaps, which were randomly taken between 0 and 0.5, namely, F1 = uniform (0,0.5), F2 = F3 = F4 = F5 = F6 = Uniform (0,1).

In cyclic Event 2, G1 = G2 = G3 = G4 = 0.5 was set, which meant that after every intervention (ST, SCL, SCU, and IM), the absorption rate of workers was only 0.5.

In cyclic Event 6, S = 10, which meant that in the absence of training, testing, or other recall, the workers’ memory strength value was 10; Z = 0.9, which meant that the rate of memory decay after the following intervention was 90% that of the previous intervention.

Table 1 shows the combined results of each correlation coefficient after meta-analysis.

In addition, we needed to normalize the coefficients of system dynamics equation in the simulation model; the results are shown in Table 2 [27].

The initial SCA, SCO, and SM values in Event 6, SCA0, SCO0, and SM0, calculated using the system dynamics equation between each SBPV and corresponding intervention factors, were 0.37, 0.5 and 0.064, respectively.

### 4.2. Result Analysis

Solving the two simulation models determined the variation trend charts of each implicit factor and safety behavior over time under active and passive interventions, respectively. The value of the disturbance factor was a random number between 0 and 1; therefore, the variation trend of the explicit factors directly affected by it over time is shown as a disordered scatter plot between 0 and 1, which is not shown here.

#### 4.2.1. Active Intervention

**Figure 5:**
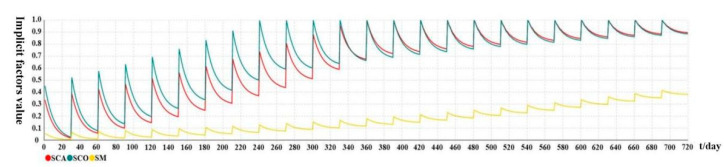
Variation trend of each implicit attitude over time under active intervention.

Figure 5 shows the variation trend of each implicit attitude over time under active intervention. It can be seen that when t = 0, the first intervention was applied to the system, and each implicit factor reached its maximum value at this stage (SCA = 0.37, SCO = 0.5, and SM = 0.064). Then, with the increase in days, under the attenuating effect of the memory forgetting curve, when t = 30, each implicit factor reached the minimum value at this stage (SCA = 0.018, SCO = 0.025, and SM = 0.003). When t = 31, the second intervention was triggered, and the effect of the intervention was superimposed with the attenuated residual value at the end of the first intervention. In addition, the attenuation trend of the second intervention stage (from the 31st to the 60th day) is slower than that of the first intervention stage. This is because the increase in the number of interventions and the strength of memory made the attenuation rate of the second intervention 90% that of the first. The changes in later interventions are similar. Among them, when t = 240, i.e., at the 9th intervention, the value of SCO became greater than 1, triggering Event 5 and making its value 1. Subsequent interventions and changes to SCA are similar. Ultimately, although the system was limited to a value of less than 1, with the increase in the number of interventions, memory strength was enhanced and the attenuation rate slowed down; all the implicit factors eventually remained at a high level (when t = 720, SCA = 0.895, SCO = 0.885, and SM = 0.381). At this time, the implicit security attitude influenced by implicit factors also remained high.

**Figure 6:**
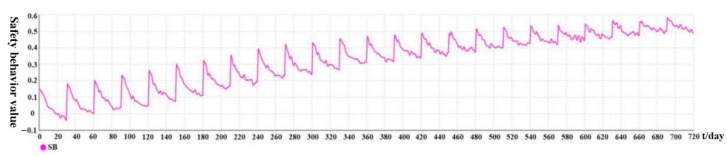
Variation trend of safety behavior over time under active intervention.

Figure 6 shows the variation trend of safety behavior over time under active intervention. It can be seen that the variation trend of safety behavior over time is similar to that of implicit factors. Higher values are at the beginning of each intervention phase, and lower values are at the end of each intervention phase. For example, from the 61st to the 90th day, when t = 90, SB = 0.039; when t = 82, the minimum value occurs (SB = 0.025). From the 451st to the 480th day, when t = 451, SB = 0.463; when t = 453, the maximum value occurs (SB = 0.495). The initial value of this stage is not the maximum value here because the safety behavior is also affected by explicit factors. The disturbance factor limits the explicit factor, whereas the value at this stage is randomly generated and highly uncertain. In addition, at the initial stage of the system, the level value of safety behavior is low, and each implicit factor is also at a low level (SB < 0); thus, the effect of explicit factors is evident at this time. However, with the increase in the number of interventions, the safety behavior level will remain high (SB ≈ 0.5). At this time, the implicit factor is relatively stable and its value is high; therefore, its effect is more evident than the explicit factor. It also shows that implicit safety attitudes have a better effect on safety behavior.

#### 4.2.2. Passive Intervention

**Figure 7:**
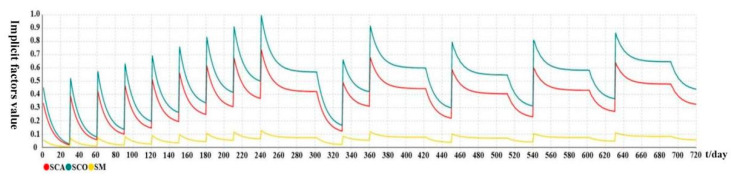
Variation trend of each implicit attitude over time under passive intervention.

**Figure 8:**
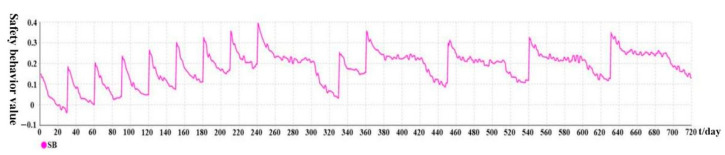
Variation trend of safety behavior over time under passive intervention.

Figure 7 and Figure 8 show the variation trends of implicit attitude and safety behavior over time under the passive intervention, respectively. The variation curves are similar in the first 240 days compared with active intervention. Indeed, at the end of each intervention stage in the passive intervention simulation model, the safety behavior value is less than 0.2, triggering a new round of intervention. Nevertheless, when t = 270, SB = 0.248 < 0.2, the passive intervention mechanism (Event 4) is not triggered at this time, and the current attenuation trend continues for 30 days. When t = 300, although SB = 0.205, and because workers have not been interceded for a long time and the values of SB remain at a low level, they are prone to slack off and trigger the complete forgetting mechanism (Event 7), leading to recovery of the decay curve rate to the initial level. When t = 330, SB = 0.032, a new intervention is triggered. The subsequent changes follow this pattern. Finally, we can see that, even with the increase in the number of passive interventions, each implicit attitude will always fluctuate up and down, and will not remain at a high level, resulting in the safety behavior level value not maintaining a high level. It is reflected that the active intervention effect is stronger than the passive intervention effect, and the implicit safety attitude effect is stronger than the explicit safety attitude from the side. The safety management of many units is still experience-based post-management with reward–punishment methods, which is not scientific.

## 5. Conclusions

This study adopted relevant psychological knowledge to establish a dual-attitude theoretical model of construction workers’ unsafe behavior, which enriches and develops the causes and prevention theories of unsafe behaviors of construction workers and provides new ideas for preventing and controlling unsafe behaviors. The authors designed the active and passive intervention system dynamics models to further understand the dual safety attitude of workers in the activation of actual work attitudes. Moreover, meta-analysis, a mathematical–statistical method, was first used to combine the correlation coefficients of previous studies as the coefficients of system dynamics equation in the simulation model, which increased the sample size and improved the statistical test efficiency. Then, the parameters of the system dynamics models were initialized, and the models were run. The closely interdependent relationship among the influencing factors was studied systematically, and the variation trend of the safety behavior and related influencing factors with time have been displayed dynamically. It will be conducive for the construction industry in the safety management of identifying workers’ unsafe behavior and the selection of more reasonable and effective intervention countermeasures. Furthermore, it will be conducive to prevent the unsafe behavior of construction workers and reduce human-caused accidents. The main conclusions are presented below:

(1) Implicit safety attitudes have a significant impact on construction workers’ unsafe behavior; therefore, managers should pay attention to improving the positive effects of implicit safety attitudes of construction workers. It can be seen from the simulation model that only when the implicit factors are enhanced to a certain extent and maintained at a high level can the safety behavior value be held at a high level. This is because implicit safety attitudes have characteristics of automatic activation and rapid response, whereas explicit factors such as unsafe physiology are characterized by considerable randomness and significant variability. Under the simultaneous action of both, highly stable implicit factors have a more significant influence on safety behavior. Managers should take effective intervention measures (safety training, communicating and fostering a safe atmosphere between workers and between workers and managers, publicizing safety culture, improving incentive mechanisms, etc.) to improve the safety capability, safety consciousness, and safety motivations of construction workers, to improve their implicit safety attitudes.
(2) The effect of active interventions is stronger than that of passive interventions, and managers should prioritize regular interventions and maintain them over time. The simulation results show that in the active intervention model, with the increase in the number of regular interventions, the safety behavior value can be kept at a high level when the implicit factors are enhanced to a certain extent. In the passive intervention model, managers only intervene when they find that the level of workers’ safety behavior is low. The effect of passive intervention is not ideal, leading to the fact that the implicit factors cannot be at a high level, and indicating that the safety behavior value, which is in a state of fluctuation, cannot reach a high level. In the actual situation, most managers often take intervention measures for workers after finding that the frequency of unsafe behavior of workers is too high or when workplace accidents occur. The former case cannot effectively prevent the occurrence of accidents; in the latter case, it will cause casualties and property damage, and employers will invest more time and energy to intervene with workers.

The developed theory and model demonstrate its potential for providing a better understanding of the cognitive processes of construction workers’ safety behaviors and serve as a powerful test bed to examine the effectiveness of interventions before implementation. However, there are still some challenges to be addressed in future research. For example, further empirical studies are needed to support the validity and applicability of the developed models, and future studies should take the negligence of the design aspect and supervision aspect into account.

## Figures and Tables

**Table 1:** Combined results of each correlation coefficients.

Path Name	K	N	r	r_c_	95%CI
ST→SCA	1	267	0.74	*	*
SCL→SCO	2	513	0.443; 0.37	0.411	(0.339, 0.483)
SCU→SCO	1	297	0.437	*	*
IM→SM	2	518	0.108; 0.15	0.128	(0.043, 0.213)
SCA→SB	5	1523	0.636; 0.301; 0.56; 0.301; 0.12	0.432	(0.392, 0.472)
SCO→SB	4	1322	0.423; 0.698; 0.19; 0.19	0.474	(0.433, 0.515)
SM→SB	8	2414	0.416; 0.567; 0.355; 0.796;0.271; 0.537; 0.31; 0.14	0.553	(0.525, 0.581)
USPH→SB	1	267	−0.14	*	*
USPS→SB	3	801	−0.387; −0.332; −0.24	−0.325	(−0.263, −0.387)
WE→SB	2	662	−0.292; −0.11	−0.174	(−0.101, −0.247)
PH→USPH	2	564	0.54; 0.710	0.650	(0.602, 0.698)
PF→USPH	2	564	0.77; 0.697	0.744	(0.707, 0.781)
WP→USPS	3	806	0.644; 0.48; 0.742	0.666	(0.627, 0.705)
LP→USPS	2	534	0.40; 0.734	0.657	(0.608, 0.706)
PW→WE	2	747	0.55; 0.67	0.635	(0.592, 0.678)
WI→WE	2	747	0.58; 0.56	0.569	(0.519, 0.617)

* indicates that this relationship only included one study. According to Valentine et al. [32], at least two studies are needed to conduct a meta-analysis, but only one was available; however, we still list it for further discussion.

**Table 2:** Coefficients of system dynamics equation after normalization.

Number	Path Name	Coefficients	Number	Path Name	Coefficients
1	ST→SCA	0.74	9	SCL→SCO	0.619
2	IM→SM	0.128	10	SCU→SCO	0.381
3	SCA→SB	0.217	11	PH→USPH	0.466
4	SCO→SB	0.207	12	PF→USPH	0.534
5	SM→SB	0.440	13	WP→USPS	0.605
6	USPH→SB	−0.012	14	LP→USPS	0.395
7	USPS→SB	−0.086	15	PW→WE	0.527
8	WE→SB	−0.038	16	WI→WE	0.473

Except for paths numbered 1 and 2, the coefficients of other paths were normalized because there is only one dependent variable for safety capability and safety motivation.

## Data Availability

Not applicable.

## Appendix A

The properties of Event 1:

Trigger type: Timeout;

Mode: Cyclic;

First occurrence time: 0 days;

Recurrence time: 1 days;

Action:

PH1=F1;

PF1=F2;

WP1=F3;

LP1=F4;

PW1=F5;

WI1=F6.

(Note: F*_i_* (*i* = 1, 2, …, *n*) is a random constant between 0 and 1.)

The properties of Event 2:

Trigger type: Timeout;

Mode: Cyclic;

First occurrence time: 30 days;

Recurrence time: 30 days;

Action:

ST1=G1;

SCL1=G2;

SCU1=G3;

IM1=G4.

(Note: G*_i_* (*i* = 1, 2, …, *n*) are constants between 0 and 1, indicating the intervention effect.)

The properties of Event 3:

Trigger type: Timeout;

Mode: Cyclic;

First occurrence time: 31 days;

Recurrence time: 30 days;

Action:

ST1=0;

SCL1=0;

SCU1=0;

IM1=0.

The properties of Event 4:

Trigger type: Timeout;

Mode: Cyclic;

First occurrence time: 30 days;

Recurrence time: 30 days;

Action:

if (SB < 0.2){

ST1=G1;

SCL1=G2;

SCU1=G3;

IM1=G4.}

The properties of Event 5:

Trigger type: Timeout;

Mode: Cyclic;

First occurrence time: 30 days;

Recurrence time: 30 days;

Action:

if (SCA>1){

SCA=1;}

if (SCO>1){

SCO=1;}

if (SM>1){

SM=1.}

The properties of Event 6:

Trigger type: Timeout;

Mode: Cyclic;

First occurrence time: 0 days;

Recurrence time: 1 days;

Action:

if(time()<30){

SCAD1=SCA0*pow(Math.E,(-time())/S)-SCAD0*pow(Math.E,(-(time()+1)/S));

SCOD1=SCO0*pow(Math.E,(-time())/S)-SCOD0*pow(Math.E,(-(time()+1)/S));

SMD1=SM0*pow(Math.E,(-time())/S)-SMD0*pow(Math.E,(-(time()+1)/S));

(Note: The above expressions are the attenuation effect from the first intervention to the second intervention; SCA0, SCO0, and SM0 are the initial values of SCA, SCO, and SM, respectively; the first term of each formula is the value of each implicit factor after the attenuation of Day *t* (*t* = 0, 1, …, *n*), and the second formula is the value after the attenuation of Day *t* + 1.)

}else{	
	if(PA2==false)

(Note: PA2 is Boolean-type, and the initial value is false. Its purpose is to control the attenuation rate between the intervention of Number H + 1 and Number H + 2 (H = 1, 2, …, *n*).)

{if(ST==G1){	
	SCA1=SCA;
	SCO1=SCO;
	SM1=SM;
	PA1=time();
	PA3++;

(Note: PA1 and PA3 are both Double-type, and their initial values are both 0. PA1 is related to time, and PA3 is related to the number of interventions. When the attenuation rate changes each time, the initial values of each implicit factor and the starting point of attenuation time during this period of attenuation duration should be reassigned again. Additionally, the intervention times should be increased by 1.)

SCAD1=SCA1*pow(Math.E,(-(time()-PA1))/S)*pow(Z,PA3)-SCA1*pow(Math.E,(-(time()+1-PA1)/S))*pow(Z,PA3);

SCOD1=SCO1*pow(Math.E,(-(time()-PA1))/S)*pow(Z,PA3)-SCO1*pow(Math.E,(-(time()+1-PA1)/S))*pow(Z,PA3);

SMD1=SM1*pow(Math.E,(-(time()-PA1))/S)*pow(Z,PA3)-SM1*pow(Math.E,(-(time()+1-PA1)/S))*pow(Z,PA3);

PA2=true;

(Note: These formulas are the attenuation effect between the intervention of Number H+1 and Number H + 2 (H = 1, 2, …, *n*).)

}else{
SCAD1=SCA0*pow(Math.E,(-time())/S)-SCA0*pow(Math.E,(-(time()+1)/S));
SCOD1=SCO0*pow(Math.E,(-time())/S)-SCO0*pow(Math.E,(-(time()+1)/S));
SMD1=SM0*pow(Math.E,(-time())/S)-SM0*pow(Math.E,(-(time()+1)/S));}

(Note: These formulas are the attenuation effect between the first and second interventions.)

}else{	
	if(ST==G1){
	SCA1=SCA;
	SCO1=SCO;
	SM1=SM;
	PA1=time();
	PA3++;

SCAD1=SCA1*pow(Math.E,(-(time()-PA1))/S)*pow(Z,PA3)-SCA1*pow(Math.E,(-(time()+1-PA1)/S))*pow(Z,PA3);

SCOD1=SCO1*pow(Math.E,(-(time()-PA1))/S)*pow(Z,PA3)-SCO1*pow(Math.E,(-(time()+1-PA1)/S))*pow(Z,PA3);

SMD1=SM1*pow(Math.E,(-(time()-PA1))/S)*pow(Z,PA3)-SM1*pow(Math.E,(-(time()+1-PA1)/S))*pow(Z,PA3);

(Note: These formulas are the attenuation effect between the intervention of the Number H + 2 and the Number H + 3 (H = 1, 2, …, *n*).)

}else{
SCAD1=SCA1*pow(Math.E,(-(time()-PA1))/S)*pow(Z,PA3)-SCA1*pow(Math.E,(-(time()+1-PA1)/S))*pow(Z,PA3);
SCOD1=SCO1*pow(Math.E,(-(time()-PA1))/S)*pow(Z,PA3)-SCO1*pow(Math.E,(-(time()+1-PA1)/S))*pow(Z,PA3);
SMD1=SM1*pow(Math.E,(-(time()-PA1))/S)*pow(Z,PA3)-SM1*pow(Math.E,(-(time()+1-PA1)/S))*pow(Z,PA3);

(Note: These formulas are the attenuation effect between the intervention of Number H+1 and Number H + 2 (H = 1, 2, …, *n*).)

		}
	}	
}		

The properties of Event 7:

Trigger type: Timeout;

Mode: Cyclic;

First occurrence time: 0 days;

Recurrence time: 1 days;

Action:

if(time()-PA1>60){

PA1=time();}
